# Increased frequency of the k-*ras* G12C mutation in MYH polyposis colorectal adenomas

**DOI:** 10.1038/sj.bjc.6601747

**Published:** 2004-03-30

**Authors:** S Jones, S Lambert, G T Williams, J M Best, J R Sampson, J P Cheadle

**Affiliations:** 1 Institute of Medical Genetics, University of Wales College of Medicine, Heath Park, Cardiff CF14 4XN, UK; 2 Department of Pathology, University of Wales College of Medicine, Heath Park, Cardiff CF14 4XN, UK

**Keywords:** k-*ras*, MYH, colorectal adenoma, G : C → T : A mutations, G12C

## Abstract

Colorectal tumours from MYH polyposis patients display an excess of somatic G : C → T : A transversions in the adenomatous polyposis coli gene. Here, we identify k-*ras* mutations in nine out of 54 (16.7%) MYH polyposis tumours. Their presence was associated with increased dysplasia and tubulovillous morphology (*P*=0.005). G : C → T : A transversions in k-*ras* were significantly more frequent in MYH polyposis adenomas than in sporadic or familial adenomatous polyposis-associated tumours (*P*⩽0.002), and all resulted in a glycine-to-cysteine substitution at codon 12.

MYH polyposis is an autosomal recessive multiple colorectal adenoma and carcinoma disorder caused by inherited defects in the *MYH* gene (Al-Tassan *et al*, 2002; Jones *et al*, 2002; Sampson *et al*, 2003; Seiber *et al*, 2003). MYH functions as a base excision repair (BER) DNA glycosylase that excises adenines misincorporated opposite 8-oxo-7,8-dihydro-2′-deoxyguanosine (8-oxoG), one of the most stable products of oxidative DNA damage (reviewed in Cheadle and Sampson, 2003). Inactivation of the *Escherichia coli* homologue of MYH leads to G : C → T : A mutations and adenomas from MYH polyposis patients display a significant excess of G : C → T : A transversions in the adenomatous polyposis coli (*APC*) gene, as compared to sporadic or familial adenomatous polyposis (FAP)-associated colorectal tumours (Al-Tassan *et al*, 2002; Jones *et al*, 2002). Adenomatous polyposis coli acts as a gatekeeper for cellular proliferation in the colon and, although mutations in *APC* may initiate adenoma formation, additional somatic changes are required for progression to carcinoma (Fearon and Vogelstein, 1990). The transition from early to intermediate adenoma is often associated with point mutation in the proto-oncogene k-*ras* (Fearon and Vogelstein, 1990). Codons 12, 13 and 61 are hotspots for mutations which reduce the GTPase activity of the protein and result in constitutive signal transduction (Bos, 1989). The most frequently mutated position in sporadic and FAP-associated colorectal tumours is codon 12 (Bos, 1989). All six nucleotide changes that result in an amino-acid substitution at this position have oncogenic potential; however, there is structural, biochemical (Al-Mulla *et al*, 1999) and clinical (Cerottini *et al*, 1998) evidence to suggest that the mutations have different effects. In this study, we screened for somatic k*-ras* mutations in colorectal cancers and adenomas with varying degrees of dysplasia from patients with MYH polyposis. We found that k*-ras* was mutated at a similar stage of the adenoma-to-carcinoma pathway as seen in other colorectal tumours. All observed mutations were characteristic of a BER defect and occurred at the same nucleotide.

## MATERIALS AND METHODS

### Samples

We analysed 50 adenomas and four carcinomas from five patients harbouring biallelic germline *MYH* mutations (one Y165C homozygote, two E466X homozygotes and two Y165C/G382D compound heterozygotes). The size, morphology and degree of dysplasia of each adenoma were determined according to the criteria of Konishi and Morson (1982). DNA was extracted from tumour samples that had been microdissected from paraffin blocks. This study was approved by the Multicentre Research Ethics Committee for Wales.

### PCR amplification and automated sequencing

We amplified exons 1–4b of k-*ras* as five fragments (primer sequences can be found at http://www.uwcm.ac.uk/study/me dicine/medical_genetics/resear ch/tmg/projects/MYH3.html). Amplification products were purified by incubation with exonuclease I and shrimp alkaline phosphatase (Amersham Biosciences, Buckinghamshire). Automated sequencing was carried out using the Big Dye Terminator Cycle Sequencing kit version 3.1 (Applied Biosystems, Cheshire). Sequencing reactions were purified using the Montage SEQ_96_ Sequencing Reaction Cleanup kit (Millipore, Hertfordshire), and analysed on an ABI PRISM 3100 Genetic Analyser. All mutations were confirmed by sequencing at least two independent PCR products.

### Somatic k-*ras* mutation database and statistical analysis

We reviewed literature reports of characterised somatic k-*ras* mutations in colorectal tumours. In total, data on 630 somatic mutations from 25 studies were included (http://www.uwcm.ac.uk/study/me dicine/medical_genetics/resear ch/tmg/projects/MYH3.html). We carried out statistical analyses using the Fisher's exact test.

## RESULTS

Using direct PCR product sequencing, we screened the k-*ras* open reading frame for somatic mutations in 18 adenomas displaying mild dysplasia, 30 with moderate dysplasia, two with severe dysplasia and four carcinomas that were surgically removed from five patients with biallelic germline *MYH* mutations. In total, we identified oncogenic mutations in nine out of 54 (16.7%) colorectal tumours (Table 1

**Table 1:** Comparison between the frequency of k-*ras* mutations and degree of dysplasia, morphology and size of colorectal tumours from MYH polyposis patients (morphology and size for adenomas only)

	**% K-*ras* codon 12 mutation**
*Dysplasia grade*	
Mild	5.5 (1/18)
Moderate	23.3 (7/30)
Severe	50 (1/2)
Carcinoma	0 (0/4)
	
*Morphology*	
Tubular	6.3 (2/32)
Tubulovillous	41.2 (7/17)
Villous	0 (0/1)
	
*Size (mm)*	
<5	0 (0/9)
5–<10	14.3 (3/21)
10–< 15	30 (3/10)
⩾15	30 (3/10)

We analysed 50 adenomas and four carcinomas for mutations in exon 1, which includes the mutational hotspots at codons 12 and 13. In all, 51, 46, 49 and 50 tumours were screened for variants in exons 2, 3, 4a and 4b, respectively. Figures in parentheses indicate the number of mutations and the total number of tumours screened.

). The frequency of k-*ras* mutations increased with the degree of dysplasia, from 5.6% (one out of 18) in mildly dysplastic adenomas to 23.3% (seven out of 30) in moderately dysplastic and 50% (one out of two) in severely dysplastic adenomas. We did not identify any activating mutations in the carcinomas. Mutations were significantly more frequent in adenomas with a tubulovillous (seven out of 17, 41.2%) compared to tubular (two out of 32, 6.3%) morphology (*P*=0.005), and tended to be more frequent in those ⩾10 mm in size (six out of 20, 30%) compared to those <5 mm in size (zero out of nine, 0%) (*P*=0.08).

**Figure 1:**
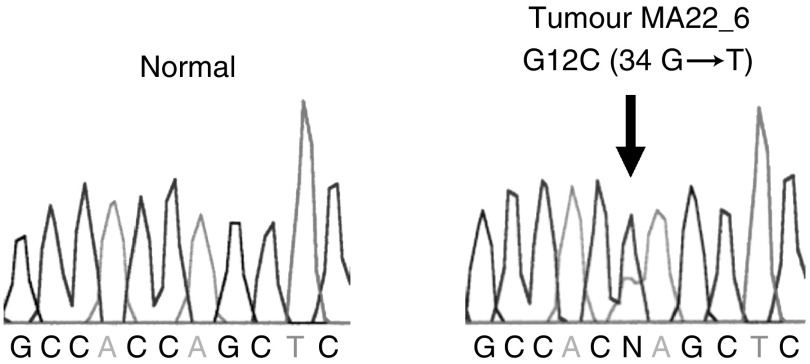
Identification of the somatic k-*ras* codon 12 cysteine mutation (G → T at nucleotide 34) in a moderately dysplastic MYH polyposis colorectal adenoma (tumour MA22_6). Sequences are shown in the reverse direction and the arrow indicates the position of the mutation.

All nine k-*ras* mutations in MYH polyposis adenomas were an identical G : C → T : A transversion at nucleotide 34, which was predicted to replace the glycine at residue 12 with cysteine (G12C) (Figure 1

). To determine whether the spectrum of mutations observed in MYH polyposis colorectal tumours was different from that seen in sporadic and FAP-associated colorectal tumours, we compiled a database of previously reported somatic k-*ras* codon 12 mutations (Table 2

**Table 2:** Spectrum of published k-*ras* codon 12 mutations in sporadic, FAP-associated and MYH polyposis colorectal tumours

	**Frequency of codon 12 mutations (%)**					
**Tumour type**	**Ala (GCT)**	**Arg (CGT)**	**Asp (GAT)**	**Cys (TGT)**	**Ser (AGT)**	**Val (GTT)**
Sporadic adenomas (*n*=182)	4.4 (8)	8.2 (15)	31.9 (58)	13.2 (24)	10.4 (19)	31.9 (58)
Sporadic carcinomas (*n*=367)	9 (33)	1.6 (6)	35.4 (130)	13.9 (51)	14.5 (53)	25.6 (94)
FAP adenomas (*n*=58)	5.2 (3)	3.4 (2)	37.9 (22)	19 (11)	8.6 (5)	25.9 (15)
FAP carcinomas (*n*=23)	0 (0)	0 (0)	65.2 (15)	17.4 (4)	8.7 (2)	8.7 (2)
MYH polyposis adenomas (*n*=9)	0 (0)	0 (0)	0 (0)	100 (9)	0 (0)	0 (0)

The cysteine mutation is significantly more frequent in MYH polyposis than in sporadic or FAP-associated adenomas (*P*⩽0.002). The actual number of mutations is indicated in parentheses.

). In total, we retrieved data on 182 and 367 somatic mutations in sporadic adenomas and carcinomas, and 58 and 23 somatic mutations in FAP-associated adenomas and carcinomas, respectively. We found a significant excess of G : C → T : A transversions in MYH polyposis tumours as compared to both sporadic and FAP-associated adenomas and carcinomas (*P*⩽0.001 and *P*⩽0.002, respectively). G : C → T : A transversions at both the first (34 G → T) and second (35 G → T causing G12V) nucleotides of codon 12 are oncogenic and are frequent in both sporadic and FAP-associated colorectal tumours. The presence of G12C and lack of G12V in MYH polyposis adenomas compared with other colorectal adenomas was highly significant (*P*⩽0.002).

## DISCUSSION

In this study, we identified somatic k-*ras* mutations in 18% (nine out of 50) of colorectal adenomas from MYH polyposis patients, which is similar to the frequency in FAP-associated adenomas (Ichii *et al*, 1993). Our data support the reported role of p21^ras^ oncogenesis in progression along the adenoma to the carcinoma pathway (Fearon and Vogelstein, 1990), since the k*-ras* mutation frequency increased from 5.6% in mildly dysplastic adenomas to 23.3% in moderately dysplastic adenomas, the frequency of k-*ras* mutation increased with villous content and, the highest proportion of mutations was observed in adenomas greater than 10 mm in size.

The significant over-representation of k-*ras* G : C → T : A transversions in MYH polyposis tumours as compared to sporadic and FAP-associated colorectal tumours is consistent with the mutational mechanism of MYH-associated tumorigenesis revealed in our previous studies (Al-Tassan *et al*, 2002; Jones *et al*, 2002). However, although there are two sites at codon 12 of k-*ras* at which G : C → T : A transversions are oncogenic, all of the observed mutations were at nucleotide 34, leading to a glycine-to-cysteine substitution. This may reflect an increased susceptibility to oxidative damage at the first base of codon 12 or repair enzyme specificity. Different mutations at codon 12 of k-*ras* are thought to have different biological effects and Cerottini *et al* (1998) reported that G12C is associated with reduced survival in patients with colon cancer. Although the somatic genetic changes that effect prognosis are likely to be complex, these clinical findings are consistent with biochemical and structural data which demonstrate that the GTPase activity, affinity for GTP and interactions with other proteins vary, depending upon the precise nature of mutations affecting k-*ras* (Al-Mulla *et al*, 1999). Further studies are therefore warranted to determine whether the specific targeting of k-*ras* in the adenomas of patients with MYH polyposis influences later clinical outcome.
